# Integrin‐associated molecules and signalling cross talking in osteoclast cytoskeleton regulation

**DOI:** 10.1111/jcmm.15052

**Published:** 2020-02-11

**Authors:** Lingbo Kong, Biao Wang, Xiaobin Yang, Baorong He, Dingjun Hao, Liang Yan

**Affiliations:** ^1^ Hong‐Hui Hospital Xi'an Jiaotong University College of Medicine Xi'an China

**Keywords:** cytoskeleton, integrin, osteoclast, podosomes

## Abstract

In the ageing skeleton, the balance of bone reconstruction could commonly be broken by the increasing of bone resorption and decreasing of bone formation. Consequently, the bone resorption gradually occupies a dominant status. During this imbalance process, osteoclast is unique cell linage act the bone resorptive biological activity, which is a highly differentiated ultimate cell derived from monocyte/macrophage. The erosive function of osteoclasts is that they have to adhere the bone matrix and migrate along it, in which adhesive cytoskeleton recombination of osteoclast is essential. In that, the podosome is a membrane binding microdomain organelle, based on dynamic actin, which forms a cytoskeleton superstructure connected with the plasma membrane. Otherwise, as the main adhesive protein, integrin regulates the formation of podosome and cytoskeleton, which collaborates with the various molecules including: c‐Cbl, p130^Cas^, c‐Src and Pyk2, through several signalling cascades cross talking, including: M‐CSF and RANKL. In our current study, we discuss the role of integrin and associated molecules in osteoclastogenesis cytoskeletal, especially podosomes, regulation and relevant signalling cascades cross talking.

## 1 INTRODUCTION

Cytoskeletal structures grant cells virous abilities including: adhere surroundings, protrusion, migration, as well as invading into tissues.^1^ These cellular functions granted by several micro‐protrusive structures of cellular cytoskeleton including: filopodia^2^ and invadosomes^3^ (podosomes in physiological and invadopodia in pathophysiological aspect, respectively) lamellipodia.^4^ In that, podosomes are rich in monocytic cell lineage such as: dendritic cell,^5^ monocyte,^6^ macrophage^7^ and osteoclast.^8^ Podosomes thus constitute the essential organelle of the monocytic actin cytoskeletal organ.^9^

**Figure 1:**
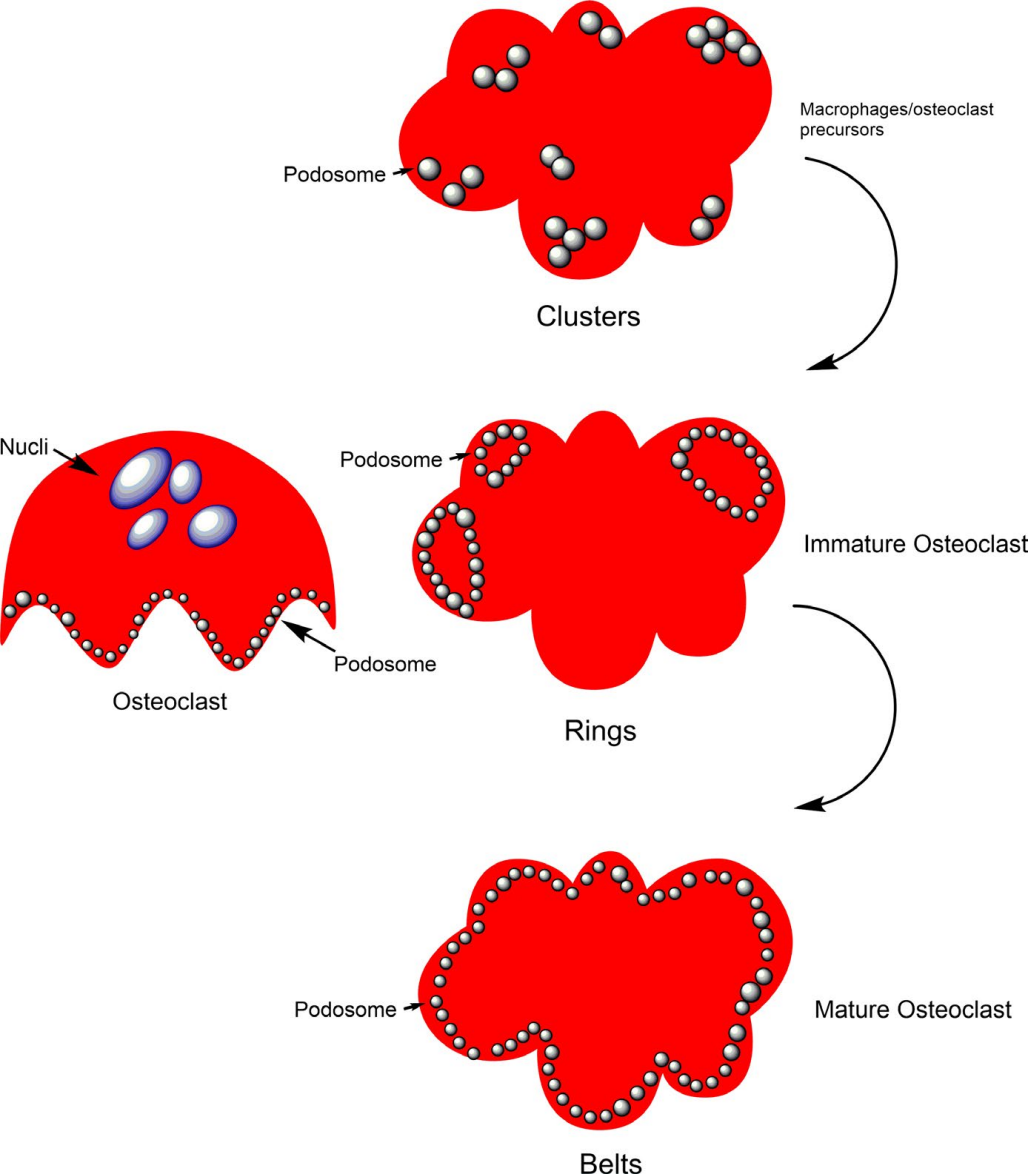
Podosome patterns during osteoclast differentiation

Osteoclast podosome is a kind of dynamic organelle, which has a definite functional role in bone metabolism and present a more specialized entity.^10^ When osteoclasts matured in vitro, the single podosome shows significantly rearranging and transforming from single podosome to podosome ‘clusters’, ‘rings’ and ‘belt’^11^ (Figure 1). When podosomes come into contact with bone, the podosomes formed in a closely packed array on the bone surface, and they are more closely connected to each other and form closed areas, which is crucial for the bone absorption characteristics of osteoclasts.^12^ Osteoclasts have the ability to degrade substrates, but their main functions are different. Rather than the enclosed area itself regulating the degradation of mineralized matrix, the bone resorption area is delineated in a tightly sealed chamber called an absorption pit.^13^ This chamber is isolated from the extracellular environment and highly acidic, thereby promoting bone resorption through the activity of cathepsin K.^14^

Currently, studies are widely conducted for exploring two issues for podosome and its cytoskeletal functions in osteoclastogenesis, such as: signalling cascades involved in the osteoclastogenesis cytoskeleton formation, and the adhesive signalling molecules involved in the physiological and pathological process of cytoskeleton (especially podosomes) regulation, in that our current review specifically covering from the study of integrin‐associated molecules and related signalling cascades cross talking including: macrophage colony‐stimulating factor (M‐CSF), receptor activator of nuclear factor‐kappaB ligand (RANKL) and phosphoinositide 3‐kinases (PI3K).

## 2 ACTIN STRUCTURE OF PODOSOMES WITH INTEGRIN ASSOCIATION

**Figure 2:**
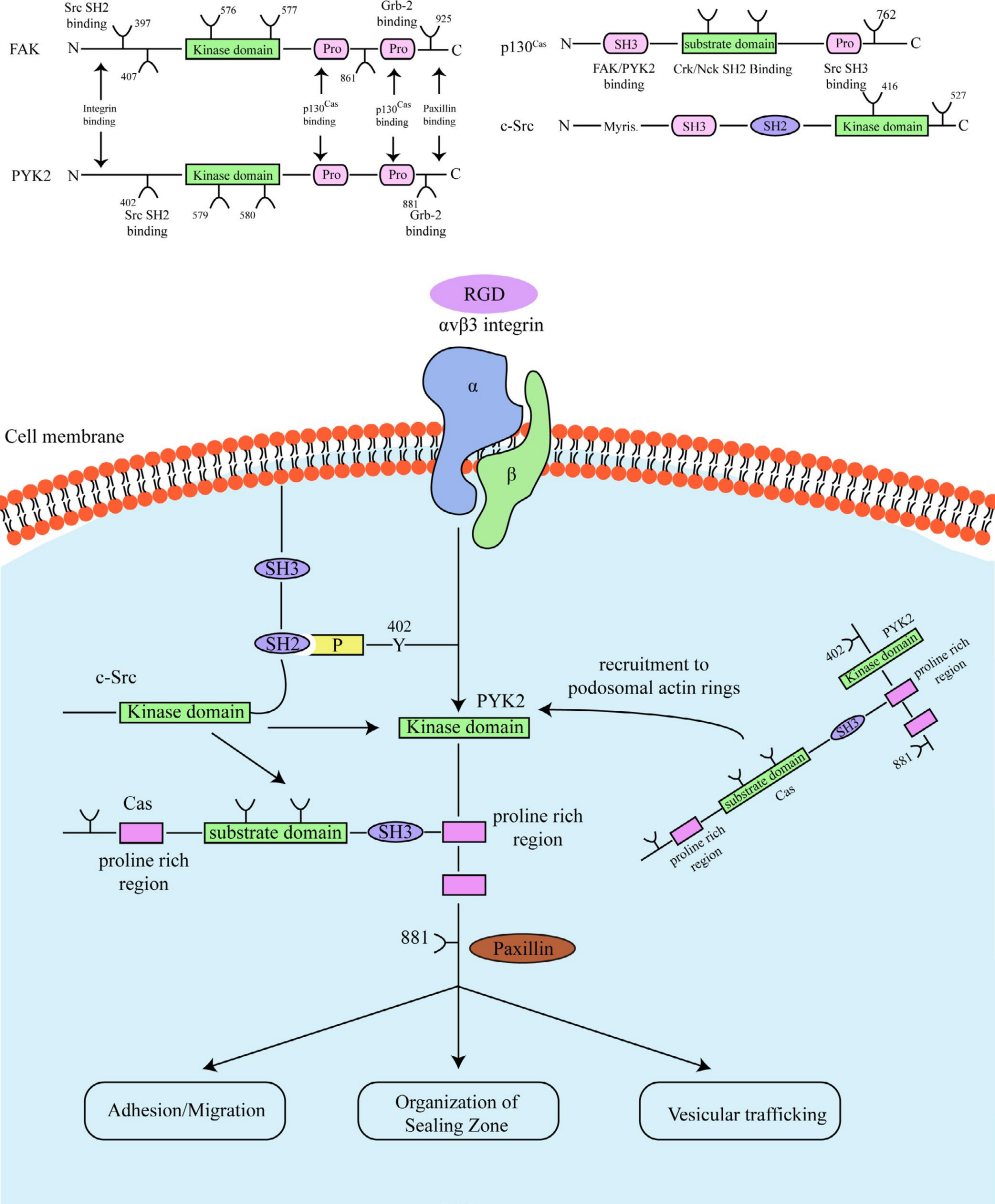
Integrin‐associated molecules structure and signalling

Podosomes have typical morphological and structural characteristics. They proposed a punctate pattern, a 0.5−1.0 μm diameter localizing in the plasmatic membrane, consisting mainly of filamentous actin.^15^ As a highly dynamic organelle, podosomes undergone a consistent disintegration and transformation in a short‐term (minute) range, which characterized by the quick overturn of actin in the structure.^12^ Although the podosome is amplified by actin production, in the fixed surface, the core structure grows perpendicular to the height of the lower layer approximate 0.6 μm.^15^ Further study found that the architectures of podosome are much complicated than originally thought: actin core, its characteristic is actin‐related protein 2/3 (Arp2/3) complex,^16^ which stimulated by WASp (Wiskott‐Aldrich syndrome protein) and cortactin nucleates actin filaments under the arrangement of the small GTPase Cdc42^17^, ^18^ (Figure 2). Besides, the core contains actin that likely branched, a network of unbranched filaments that connects the top of the podosome to the ventral plasma membrane.^19^ Another set of unbranched actin cables connects the podosomes to help organize them into higher order groups.^20^ The contractile nature of these cables may also contribute to the regular pattern of a typical podosome groups.^21^

Integrins and hyaluronan receptor CD44 are the main transmembrane adhesion molecule of podosomes, which is a bridge connecting internal and external.^22^ The transmembrane metalloprotease MT1‐MMP (MMP‐14) was partly via microtubule/motor protein network transmitted to the podosome basal side, which all through the transport vesicle.^23^, ^24^, ^25^ By interacting with actin filaments, the enzyme remains spatially confined to the site of podosomes formation.^12^ A number of integrin‐related structural proteins have been found around the actin core, including paxillin, vinculin and talin.^15^ Most of these molecules can be clearly located in the podosome core or ring structure, while others, such as p130Cas (Src kinase, crk‐related substrate of Cas), have not been finally located.^26^, ^27^ Integrin shows an isotype‐specific localization, with β1 integrin locating the preferred core, while both the β2 integrin and β3 integrin are localized in the ring structure.^15^, ^28^, ^29^, ^30^

Integrins are usually present low affinity, or inactive, or in basal states and could be activated from both directions.^31^ The receptor aggregation induced by the ‘outside‐in’ signal after the occupation of the integrin cell's external distributor further increases the affinity of integrin to ligand. Another essential mode of activation is ‘inside‐out’ signal cascades, including signal cascades induced by secondary receptors (such as: cytokine receptors) that transmit conformational variations in the interior cellular portion of integrin to the exterior cellular domain.^31^, ^32^ Integrins are thus prepared for ligand binding and for signalling outside the cell, further regulate cytoskeleton reorganization.^13^ However, the activity state and exact molecular mechanisms of integrins in podosomes still remain elusive. In fact, αvβ3 integrin of osteoclastic podosome belt presents a basal state, which has no responding to the monoclonal antibody against activation‐stimulated epitopes.^33^ On contrary, the activation is observed when M‐CSF treated immunoreactivity against the αvβ3 integrin.^33^

## 3 INTEGRIN SUBUNIT AND OSTEOCLAST CYTOSKELETON REGULATION

Integrins are a superfamily of cell‐surface receptors, which could conduct cellular and cell‐matrix communications or interactions.^34^ Integrin is involved in mediating signalling mechanisms that participate several cellular functions, such as: embryonic progression, cellular homeostasis, leucocyte activation and homing, coding for cell death, and the benign and malignant tumour cell growth and metastasis.^35^ The integrin membrane glycoprotein heterodimeric consists of α‐ and β‐subunit.^36^, ^37^ This adhesive molecule plays a essential role in osteoclastogenesis via regulating osteoclast adhesive ability, regulating cell migration and sealing zone to form required cytoskeletal structures.^38^ Among various integrins, β_3_ integrin is most expressed in osteoclasts,^33^ while other integrins including the vitronectin/fibronectin receptor and the collagen/laminin receptor α_2_β_1_ are also relatively low expressed in mammalian osteoclasts at lower level.^39^, ^40^

On the other hand, however, the specific molecular signalling mechanisms of integrins for its role in osteoclast activation are far from being explored. Interestingly, in deficiency of αvβ3 murine model, the amount of bone surface osteoclasts did not decrease, indicating that the lack of αvβ3 has little inhibitory effect on the number of osteoclast differentiation, that is, osteoclast caused by the lack of αvβ3. The phenomenon that cells are separated from the bone surface does not cause a decrease in the number of osteoclast differentiation.^41^, ^42^ In fact, the role of integrin, especially its subunit αvβ3 in the initially adhesive actions of osteoclasts is well‐established in several studies.^43^, ^44^, ^45^ However, the αvβ3 integrin intracellular localization remains exploring. Study has been demonstrated that the vitronectin receptor, αvβ3, was substantial in the sealing zone of matured osteoclasts. However, some studies failed to detect the αvβ3 presence in the osteoclast sealing zone membrane.^45^ Among these studies, in the matured osteoclasts the vitronectin receptor was identified to localize in the ruffled borders, intracellular vesicles and basolateral membranes. However, comparing the differences among these studies, we could conclude that these discrepancies might lies in the different study conditions such as: different brand of antibodies, various detection methods and the different osteoclasts status (activated osteoclasts, migrating osteoclasts, etc). Therefore, further studies are needed for exploring these issues.

As mentioned above, although osteoclasts express high levels of αvβ3 integrin, mammalian osteoclasts can also express other integrins at low levels, such as: the vitronectin fibronectin receptor α_v_β_1_ and collagen laminin receptor α_2_β_1_.^46^ In addition, unlike mammalian osteoclasts, the avian osteoclasts suggested could express more integrins, such as: fibronectin receptor α_v_β_1_ vitronectin receptor α_v_β_5_ and β_2_ integrins.^47^, ^48^, ^49^ However, osteoclast adhesion to bone surface involves the interaction of integrin with bone matrix extracellular matrix (ECM) proteins. Specifically, for example the murine osteoclasts adhere to ECM by the αvβ3‐dependent manner, whereas the ECM proteins comprised by Arg‐Gly‐Asp (RGD) sequences. Moreover, the Arg‐Gly‐Asp (RGD) sequences containing the bone sialoprotein, osteopontin, and cryptic RGD site in denatured collagen type I and vitronectin.^50^, ^51^, ^52^ Besides the β_3_ integrin, recently it has been demonstrated that murine osteoclasts could via α2β1 integrin to adhere the native collagen type I, which also in an RGD‐dependent manner.^53^ In addition, osteolysis could inhibited through both the anti‐β2 and anti‐β1 antibody treatment, and soluble RGD peptides could decrease the avian osteoclast adhesive abilities and osteolysis.

## 4 CRUCIAL MOLECULES OF INTEGRIN‐RELEVANT OSTEOCLAST CYTOSKELETAL REGULATION

Cellular communications and interactions with extracellular environment are achieved by the recognition between cellular transmembrane receptors and immobilized or soluble ligands, and the signalling transduction from exterior to the interior of the cell. Podosomes formation and its cytoskeletal regulating functions are the net results of complicated signals interaction and both adhesive molecules and relevant receptors participation.^23^ In fact, integrin signalling cascades involved in the osteoclastogenesis or bone cell homeostasis are mediated by various ECM molecules, which communicate with the exterior domain of integrin and further transmit the interior signals, namely ‘outside‐in signalling’. These crucial molecules includes: c‐Src,^54^ c‐Cbl,^55^ p130^Cas^ 26and proline‐rich tyrosine kinase 2 (Pyk2).^56^ Although the exact molecular mechanism of these molecules in osteoclast podosome regulation remains exploring, their role in the integrin‐relevant cellular cytoskeletal regulation is well documented. For example, in fibroblastic cells, engagement between integrins and their ligands could stimulate the autophosphorylation and activity of focal adhesion kinase (FAK). Moreover, the FAK recruit the crucial molecules such as: c‐Src.^57^ Further, in turn the interaction of c‐Src and Grb2 could cause the phosphorylation of FAK tyrosine 925. Consequently, these molecules communications and interactions will lead to the activation of signalling transmission, such as: extracellular signal‐regulated protein kinase (ERK) signalling transduction.^30^, ^57^ While the p130^Cas^ interacting with the c‐Src SH3 domain of and could be tyrosine phosphorylated by this molecule, further lead the cytoskeleton reorganization through Crk and Nck.^58^

Interestingly, c‐Src was not only found highly expressed in primary osteoclasts, but also found in osteoclasts, which derived from RANKL induced from murine macrophages linage RAW264.7 cells. This suggested that the expression of c‐Src appeared to be under the control of RANKL signalling cascades.^59^, ^60^ As a member of the non‐receptor tyrosine kinase family, c‐Src was found to be crucial molecules for osteoclast cytoskeletal construction periods, such as: osteoclastic precursors fusion and polarization, which the targeted disruption of c‐Src in mice could induce osteopetrosis. The Src^−/−^ mice have inactive osteoclasts that lack ruffled border. Besides, Src^−/−^ osteoclast precursor cells lack the capacity for spreading while the wild‐type counterpart spreads with 60 min during the in vitro cell culture. These results suggesting aforementioned molecules associated with osteoclast cytoskeletal regulation.^61^, ^62^ For example, although the absence of c‐Src is sufficient to abrogate osteolysis in vivo, there has no decreasing for osteoclast cell numbers. These contrary results also raise a speculation for other various molecules participation and compensation during osteoclast function establishment. Tanaka et al have demonstrated that comparing to the wild‐type osteoclast counterpart, the level of tyrosine phosphorylation of c‐Cbl immunoprecipitated from Src osteoclasts is significantly decreased. Further study clarified c‐Src associates and colocalizes with c‐Cbl in the osteoclast vesicles intracellular membranes.^63^

In fact, targeting abrogate the c‐Src in murine model could lead the osteopetrosis caused by the osteoclasts functional defect.^64^, ^65^ In that, Src^−/−^ murine‐derived osteoclasts demonstrated abnormal cytoskeletal structure, delayed in cell migration and subsequently decreasing the osteolysis. Moreover, in the aspect of cytoskeleton regulation, c‐Src is crucial molecules for regulating the podosome function and sealing zone formation. Specifically, the induction of αvβ3 integrin could activate c‐Src, further lead the phosphorylation of adaptor molecules and cytoskeleton‐associated kinases (c‐Cbl, Pyk2 and Crk‐associated substrate [p130Cas]) in osteoclasts.^61^, ^66^, ^67^ Besides that, c‐Src interacts with these molecules for forming a complexes, which present and localize in podosomes, participating the osteoclast skeletal formation and cellular functions including: fusion and migration.

**Figure 3:**
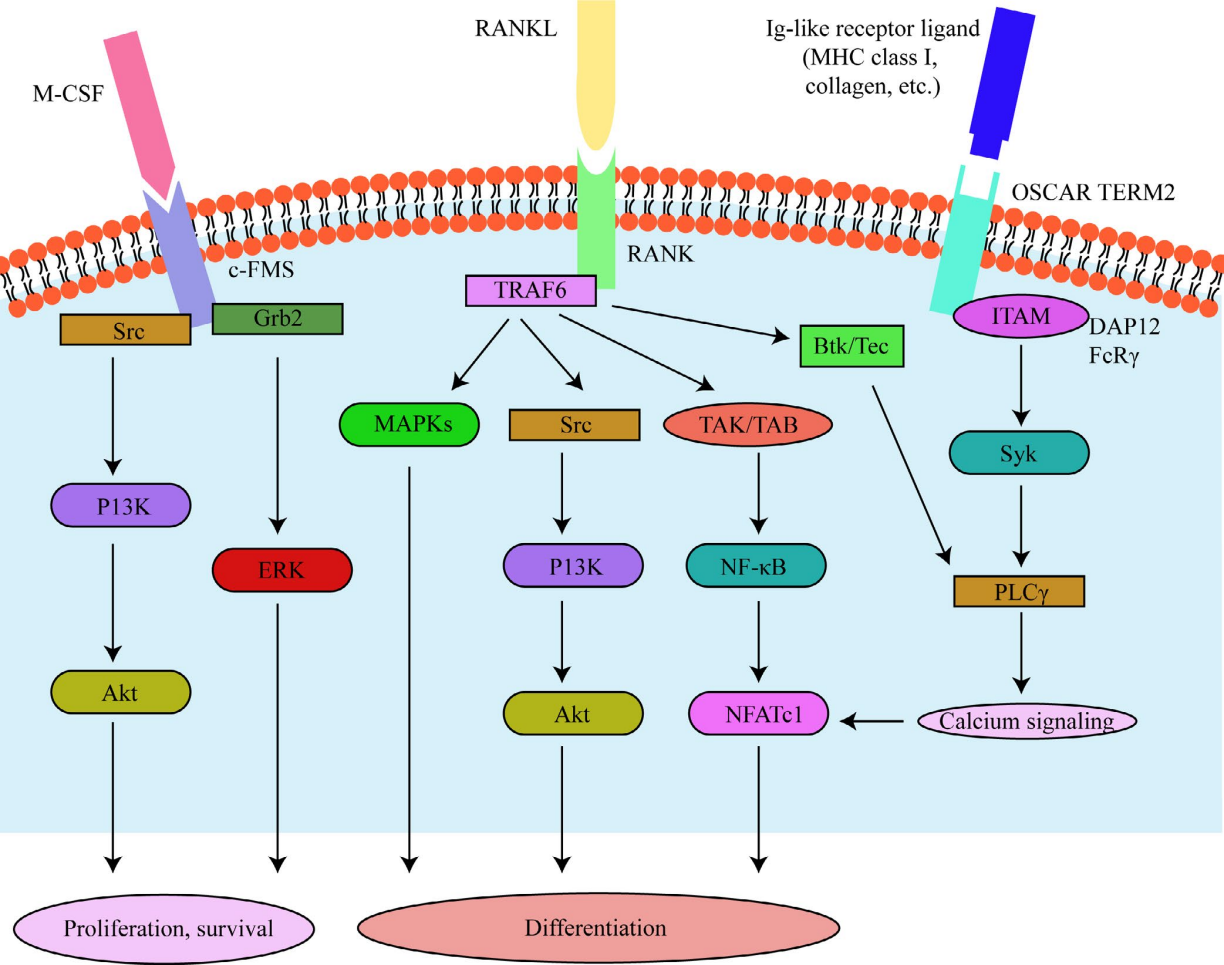
Signalling cross talking of osteoclastogenesis

Recently, Zhao et al^68^ have identified Pyk2 is a main adherent tyrosine kinase in osteoclasts. As a member of the FAK family, Pyk2 is highly contained in central nervous system (CNS) and haematopoietic cell lineage, such as: monocytes/macrophages and osteoclasts. Importantly, Pyk2 shares approximately 45% of overall amino acid identity with FAK. Therefore, Pyk2 has a high degree of conservation sequence surrounding the SH2‐ and SH3‐domain binding site.^55^ Upon osteoclast attached the skeleton, Pyk2 localizes to cytoskeletal fractions and colocalizes with the F‐actin of podosomes and podosome organelle formed sealing zones.^69^ In addition, Pyk2 kinase could also colocalize with vinculin in the podosome for its actin‐riched organelles spreading and reorganization in the forms of belt or rings in osteoclasts on glass, and in the form of sealed zone in matured functional osteoclasts on skeleton. Besides that, Pyk2 C‐terminal domain also comprised paxillin‐binding sites. Therefore, Pyk2 is closely associated with the cytoskeletal proteins recruiting, which following the integrin activation in osteoclast.^56^ Specifically, Pyk2 is suggested to play a critical role in osteoclast adhesive function‐related cytoskeletal organization, such as: osteoclastic precursors migration, osteoclast spreading and actin sealing zone formation. Similar to Pyk2, once osteoclast adheres to ECM, p130^Cas^ was clarified to be highly tyrosine phosphorylated and localized to the actin‐rich podosome in the forms of belt or rings in osteoclasts on glass, and in the form of sealed zone in matured functional osteoclasts on skeleton.^70^ However, study shown that for the c‐Src^−/−^ osteoclasts, p130^Cas^ could not phosphorylated and scattering in osteoclastic cytoplasm, suggesting c‐Src is up‐stream of p130^Cas^ molecule during its osteoclasts cytoskeletal association^71^ (Figure 3).

## 5 CRITICAL SIGNALLING AND THEIR CROSS TALKING INVOLVED IN INTEGRIN‐ASSOCIATED OSTEOCLAST CYTOSKELETAL REGULATION

### 5.1 Immune tyrosine‐based activating motif‐bearing adapters

Several studies suggested the importance of immune tyrosine‐based activating motif (ITAM)‐bearing adapters for its role in integrin signalling during the osteoclast cytoskeletal regulation.^72^, ^73^ Meanwhile, components immunoreceptor signalling might also cross talk with other signalling cascades of osteoclastogenesis, such as: RANKL signalling pathways. In that, ITAM‐bearing transmembrane adapters through a noncovalent manner complicated with these immunoreceptors. Receptor ligation could cause the ITAM tyrosines phosphorylation through Src‐family induction, which consequently via the SH‐2 domain to lead the recruitment of Syk or ZAP‐70 kinases.^66^, ^74^ Further, the activation of Syk and ZAP‐70 kinases could stimulate various downstream signalling pathways, such as: ITAM/PLCγ signalling cascades.^66^

Studies proved two critical ITAM‐bearing transmembrane adapters: DAP12^75^ and the FcRγ chain (FcRγ)^76^ have involved in the integrin signal transduction during the several cellular biology of including: neutrophils, macrophage and osteoclast. For example, combined genetic deficiency of DAP12 and FcRγ could abrogate β2‐stimulated functional reactions and downstream signalling cascades transmitting in neutrophils, which during the process without interacting with other integrin‐independent signalling cascades.^77^, ^78^ However, in macrophages, DAP12 and FcRγ are two key adapters for β2 integrin conducted ERK signalling cascades activation.^79^ Although studies showed that the double deficiency of DAP12^−/−^/FcRγ^−/−^ mutation significantly induced the osteopetrosis, the solitary DAP12^−/−^ mutation also could lead to the defective development of osteoclastic functions.^78^, ^80^ Interestingly, the double deficiency of DAP12^−/−^/FcRγ^−/−^ mutate osteoclast differentiates normally in vitro, this might lie in exitance of other ITAM‐containing receptor localizing on the osteoclast surface. However, double deficiency of DAP12^−/−^/FcRγ^−/−^ mutation could cause the failure of podosomes structure formation such as: podosome belts or sealing zone, and consequently decreasing the ability of bone resorption.^73^ Therefore, the overall outcome of the double deficiency of DAP12^−/−^/FcRγ^−/−^ mutation and the solitary DAP12^−/−^ mutation in osteoclast phenotype is very similar to the results of β3 integrins deficiency in osteoclast.^78^ Moreover, in the solitary DAP12^−/−^ mutate osteoclast precursors, cells fail to migration on αvβ3 integrin‐ligand–coated surfaces. Thus, the DAP12 and FcRγ are the critical adapters for regulating the development of osteoclast precursors and function of matured osteoclast, which speculated as the result of a interaction with αvβ3 integrin signal activation.

DAP12, as the ITAM‐bearing transmembrane adapter molecule highly, is expressed in immune cells. Besides, DAP12 is a crucial orchestrator of between integrin signalling pathways and various stimuli. For example, DAP12 could pair myeloid cellular surface‐resident receptors, such as: receptor on osteoclasts and triggering receptor expressed on myeloid (TREMs) cells. In fact, resorptive function abolished in DAP12^−/−^ osteoclast mainly caused by the αvβ3 integrin and M‐CSF signalling impaired. Specifically, as the responding to M‐CSF induction and reacting to αvβ3 integrin engagement, c‐Src leads the phosphorylation of DAP12 ITAM motif through the tyrosine residues.^81^ In that, the DAP12 cytoplasmic domain containing the ITAM motif, which is a docking site for none receptor tyrosine kinases, such as: Syk, which could be involved in the DAP12 for its role in osteoclastic cytoskeletal regulation, further regulates osteoclast bone resorptive function. Moreover, both could regulate the phosphorylation of PLCγ2 in osteoclasts. Therefore, it is suggested that FcRγ and DAP12 regulate the PLCγ2 through the responding to the engagement of αvβ3 integrin during osteoclastogenesis (Figure 1). However, the role of PLCγ2 in cellular regulation, especially the role in communication to adhesive factors, remains exploring. Studies showed modified ITAM could bind Syk then triggering signalling transduction, such as: PLCγ2, which demonstrated highly expressed in osteoclast cytoskeletal reorganization.

### 5.2 Non‐receptor tyrosine kinase Syk

Syk is required for β2 integrin conducted cellular spreading and the activation of ERK signalling in myeloid cells including osteoclast.^82^ Syk, as a non‐receptor tyrosine kinase, is essential to immune system and associated with various functions of the immune cells. Syk also is crucial component of Fc‐receptors, including: Fcε‐receptors and Fcγ‐receptors on macrophages and Fc receptor‐related collagen receptor GpVI of platelets.^83^ Syk could conduct the β_1_, β_2_ and β_3_ integrins signalling in various cells, such as: monocytes/macrophages and neutrophils.^84^ As aforementioned, most of those cellular abilities of Syk are associated to the binding with receptor‐associated tyrosine‐phosphorylated ITAMs immunoreceptors for further signalling cascades transduction. The Syk has remarkable role in several inflammatory and immune pathological processes, which presented in various diseases including musculoskeletal disorders, such as: arthritis.^84^ However, the molecular functions of Syk in osteoclastogenesis‐related diseases remain exploring.

In fact, in osteoclasts DAP12 and FcRγ activate the Syk subsequently conducted the development and function of osteoclast. In addition, Syk^−/−^ mutation osteoclastic precursors failed to differentiate to mature osteoclasts or present bone adhesive activity. Other study showed Syk could constitutively phosphorylate in stably adherent osteoclasts, and plating preosteoclasts on αvβ3 ligands could lead the phosphorylation of Syk.^74^, ^85^ Those results, importantly, clarified the interactions between bone homeostasis, especially osteoclast cellular functions, and immunoreceptor‐like signalling, therefore provided critical evidence for novel field of bone and immune system, namely ‘osteoimmunology’.^86^ In addition, these studies suggest that Syk signalling involved the regulation of osteoclast cytoskeleton and adhesive function, which might have a cross talking with integrin signallings and relevant molecules. Several studies developed Syk^−/−^ murine model, in order to clarify the role of Syk in the engagement or communication with integrin signalling pathway. However, due to the perinatal lethality in Syk*^−/−^* murine model, the study failed to test the Syk^−/−^ animals bone morphology,^87^ it is until recently studies provide the evidence for Syk role in bone homeostasis in vivo.

During our current manuscript preparation, Csete and colleagues have further reported that they accomplished the conditional abolish of the Syk, which could generate a murine model with osteoclastic‐specific Syk deficiency mic (Syk*^Δ^* ^OC^) or haematopoietic Syk deficiency mice (Syk*^Δ^* ^Haemo^) via. Subsequently, through using Cre recombinase expressed under the control of the Ctsk or Vav1 promoter, they demonstrated that the density of bone trabecular presented an increasing manner in Syk*^Δ^* ^Haemo^ and Syk*^Δ^* ^OC^ mice.^88^ In other hand, study manifested that osteoclast with the phenotype of Syk*^−/−^* could significantly resemble the osteoclast with β3 integrin‐deficient phenotype. Moreover, in Syk*^−/−^* osteoclastic precursors, cells manifested in adhesion, Vav 3 phosphorylation and spreading defects, further plate the Syk*^−/−^* osteoclast on αvβ3 integrin‐ligand–coated surface and do not resorb bone.^43^, ^89^ These novel study results demonstrated the crucial role of Syk in osteoclast associated to the β3 integrin‐mediated cellular function.

## 6 CRUCIAL SIGNALLING CROSS TALKING IN OSTEOCLAST CYTOSKELETON REGULATION

As two primary key osteoclastogenesis signals, M‐CSF and RANKL not only involved in the stimulation of osteoclastic differentiation, but also organize the cytoskeleton of matured osteoclast, thereby regulating their capacity to degrade bone, together and/or respectively.^90^, ^91^ In fact, previous studies considering that the binding of the M‐CSF and c‐Fms induced signalling pathways required for osteoclastic precursor survival and proliferation,^92^ whereas the binding of RANKL and RANK conducted signalling cascades required for differentiation of osteoclastic precursors and the resorptive function of matured osteoclast.^93^

In that, M‐CSF interact with its cognate receptor c‐Fms could lead the specific tyrosine residues autophosphorylation and transphosphorylation in the site of cytoplasmic tail of c‐Fms.^94^ However, among the c‐Fms cytoplasmic tail tyrosine residues, four crucial tyrosine residues (including: Y559, Y697, Y721 and Y921) participate the regulation of osteoclastic precursors survival and proliferation.^95^ Particularly, among these four critical tyrosine residues, phosphorylated Y559 could bind with c‐Src, subsequently the phosphorylated Y559 and c‐Src complex cause the c‐Cbl and phosphatidylinositol 3‐kinase (PI3K) recruitment, which PI3K could further activate the Akt signalling.^96^ Besides that, the phosphor‐Y697/Y974 could interact with Grb2 that stimulated ERK signalling.^97^ Recently, studies demonstrated that PI3K is also clarified localizing in podosomes via the engagement between c‐Src and gelsolin in response to αvβ3 integrin activation. In that, c‐Src could lead the phosphorylation of Y731 tyrosine residue in c‐Cbl. In fact, the Y731 tyrosine residue in c‐Cbl is known as a PI3K binding site, and the mutation of c‐Cbl/Y731 overexpression could inhibit bone resorptive activity.^98^ Other study has showed using the PI3K inhibitor wortmannin could decrease the osteoclastic adhesive ability and cause the podosomes disappearing.^99^ These results suggested that the c‐Src/PI3K/Akt signalling pathway might play a essential role in osteoclastic cytoskeleton assembling, especially for podosomes formation and motility.

Moreover, c‐Src following αvβ3 integrin engagement could directly phosphorylate Syk. Indeed, Syk SH2 motifs mutation could disrupt the molecule ability on DAP12 communication, whereas retaining interaction with αvβ3 integrin abrogates the communication of Syk and Src and therefore regulate the osteoclast cytoskeleton reorganization.^100^ Besides the αvβ3 integrin‐binding ability for osteoclastic adhesive function, Syk also associated with the M‐CSF signalling cascades in a DAP12‐dependent manner. In addition, Syk SH2 motif mutation could also defect the binding ability to the DAP12 ITAM motif and abrogate the response to M‐CSF signalling. Thus, the association of Syk SH2 motifs with DAP12 could be speculated as a critical convergence point for αvβ3 integrin and M‐CSF signalling cascades to the osteoclastic cytoskeleton regulation. However, this cellular mechanism is conducted by a autophosphorylation by Src rather than transphosphorylation.^101^

In the late stage of osteoclastogenesis, osteoclastic resorptive capacity mainly affected by its cytoskeleton reorganization.^102^ Once contact with bone surface, osteoclasts could demarcate the acidified bone matrix resorptive zone from the bone surface and apical membrane through the actin cytoskeletal reorganization to form the podosome belt, further a sealing zone, which subsequently form a gasket to restrain the lacunar acid leakage.^103^ Indeed, osteoclastic resorptive ability depends on the sealing zone and actin rings formation. Besides, vast studies for exploring the RANKL‐induced osteoclast formation form precursors.^102^, ^104^, ^105^ Studies have been also conducted to explore the osteoclast cytoskeleton regulated by RANKL. Specifically, studies have reported that the RANK signalling might associate with c‐Src, therefore suggesting the interaction between RANK and αvβ3 integrin.^63^ As aforementioned, c‐Src associated with the osteoclastic cytoskeleton regulation by activating the receptor/kinase complex. In addition, RANKL could also activate the PI3K/Akt signalling cascades through tumour necrosis factor receptor‐associated factor (TRAF), whereas the genetic deletion of c‐Src or the inhibitor of Src‐family kinase could inhibit RANKL‐stimulated osteoclast resorptive ability via decreasing the Akt activation. Suggested the cytoskeletal regulating activation of RANKL/TRAF/PI3K/Akt signalling might cross taking with Src kinase (Figure 3).

However, the ability of osteoclastic bone resorption finally achieved on the actin‐rich sealing zones formation, the cellular actin cytoskeletal integrity and consequently via a complicated signalling transduction fashion. The osteoclasts polarization could be regulated by the RANKL/RANK interact with αvβ3 integrin.^106^ Besides that, αvβ3 integrin could also up‐regulate the canonical signalling complex consisting of Syk and Pyk2 for allowing the actin rings formation in osteoclast.^56^, ^107^, ^108^, ^109^, ^110^

## CONFLICT OF INTERESTS

The authors declare that they have no competing interests.

## AUTHORS' CONTRIBUTIONS

DH, BW, LY, BH, LK involved in conception and design, analysis and interpretation of data; drafted the manuscript and revised it critically for important intellectual content; finally approved the version to be published. XY involved in acquisition of data, analysis and interpretation of data; LK conceptualized and designed, revised the manuscript critically for important intellectual content, finally approved the version to be published, accounted for all aspects of the work in ensuring that questions related to the accuracy or integrity of any part of the work are appropriately investigated and resolved. All authors read and approved the final manuscript.

## CONSENT FOR PUBLICATION

The manuscript is approved by all authors for publication.

## Data Availability

All data and materials were included in the manuscript.
